# Tactile and haptic interaction in STEM learning: a scoping review of embodied and multisensory pathways for conceptual learning processes

**DOI:** 10.3389/fpsyg.2026.1840215

**Published:** 2026-08-07

**Authors:** Ricio Milo Salibay, Mae E. Entice, Mary Jane Q. Paraiso, Vincent Gee A. Tampus, Joseph Vergil M. Espina, Achilles I. Cabaron, Cherenlie G. Apas

**Affiliations:** 1 Science Department, Cebu Technological University, Cebu, Philippines; 2 Special Needs Department, Cebu Technological University, Cebu, Philippines; 3 Technology and Livelihood Department, Cebu Technological University, Cebu, Philippines

**Keywords:** embodied cognition, haptic interaction, multisensory learning, scoping review, stem education, tactile learning

## Abstract

Conceptual learning in science, technology, engineering, and mathematics (STEM) often involves phenomena that are abstract, dynamic, and not directly observable. Instruction in many STEM classrooms continues to rely predominantly on visual representations and spoken explanations, which may limit opportunities for learners to engage with scientific concepts through multiple sensory pathways. Within this perspective, tactile and haptic interaction offers an alternative sensory pathway through which learners can explore scientific phenomena through direct sensorimotor experiences. This scoping review examines how tactile and haptic learning approaches are implemented in STEM education and how these interventions translate physical phenomena into perceptible sensory experiences that support conceptual understanding, embodied interaction, and active engagement across diverse educational contexts. Following Arksey and O'Malley's scoping review framework and reported in accordance with PRISMA-ScR guidelines, literature published between January 1, 2015 and December 15, 2025 was systematically identified, screened, and synthesized from five major databases. To strengthen the comprehensiveness of the review and broaden coverage of relevant studies published in mainstream STEM education journals, supplementary targeted searches were conducted during the revision process while maintaining the original review timeframe and eligibility criteria. A total of 23 studies met the inclusion criteria, encompassing tactile models, vibrotactile systems, embodied simulations, and force-feedback interfaces implemented across diverse educational settings. Across the reviewed studies, tactile and haptic interaction supported conceptual access to abstract phenomena, procedural engagement, active participation, and multisensory meaning-making in STEM learning. Synthesizing patterns across the included studies, this review proposes the Tactile and Haptic Interaction (THIn) conceptual model, which explains how physical phenomena are translated into sensory interaction mechanisms and pedagogical processes that support conceptual learning through embodied cognitive experiences. The findings clarify how tactile interaction functions as a sensory–cognitive pathway connecting perception, embodied action, and conceptual reasoning in multisensory STEM learning environments while situating these insights within a broader body of STEM education literature.

## 1 Introduction

Conceptual learning in science, technology, engineering, and mathematics (STEM) frequently involves phenomena that are abstract, dynamic, and not directly observable. Scientific concepts such as force, motion, wave behavior, and system dynamics often require learners to construct mental representations of processes that cannot be directly perceived. In many educational contexts, these concepts are presented primarily through visual diagrams, mathematical representations, and spoken explanations. While these approaches can be effective for some learners, they may limit opportunities to engage with scientific ideas through multiple sensory modalities.

Research in cognitive science and educational psychology has increasingly emphasized the importance of embodied cognition, suggesting that learning processes are grounded in interactions between perception, action, and conceptual reasoning. From this perspective, conceptual understanding can be supported when learners interact with representations of phenomena through sensorimotor experiences that link bodily interaction with cognitive interpretation. Multisensory learning environments can therefore provide opportunities for learners to explore abstract relationships through perceptual and physical interaction (Ambika and Vayola, 2023; Tafese and Kopp, 2025). From this perspective, conceptual understanding emerges through the coordination of perceptual experience, bodily action, and cognitive interpretation during interaction with learning environments.

The role of sensory pathways in conceptual learning becomes particularly evident for learners whose access to auditory information is limited. Deaf and hard-of-hearing (DHH) learners often encounter barriers in STEM education when instruction relies heavily on auditory explanations and sound-based demonstrations (Marshall et al., 2016). Such instructional practices can constrain opportunities for conceptual engagement when key information is communicated through auditory channels. McClive et al. (2020) report that these conditions may limit conceptual understanding and participation in STEM learning environments. Studies examining learning among DHH students highlight the importance of alternative sensory pathways, including visual and tactile interaction, for supporting reasoning and engagement with scientific ideas (Adeduyigbe et al., 2026; Ross et al., 2020; Thom and Hallenbeck, 2021).

Tactile and haptic interaction represents one pathway through which learners can engage with scientific phenomena through embodied exploration. Tactile pedagogies involve the use of structured touch-based representations—such as raised diagrams, physical models, or textured surfaces—to support conceptual understanding. Haptic interaction refers to the use of force, vibration, or motion-based feedback that allows learners to perceive information through bodily interaction with physical or virtual environments. Through these interactions, spatial relationships, dynamic processes, and physical forces become perceivable sensory experiences.

When integrated into instructional activities, tactile and haptic approaches allow learners to explore scientific phenomena through direct manipulation and sensorimotor interaction. For example, tactile models may allow learners to examine spatial structures and relationships, while vibrotactile or force-feedback systems can represent dynamic processes such as motion, resistance, or wave behavior. These sensory experiences can provide perceptual cues that help learners construct mental representations of abstract relationships between physical variables.

Research on tactile and haptic learning environments spans several interdisciplinary domains, including science education, human–computer interaction, cognitive psychology, and assistive technology design (Constantinou et al., 2020; Mirzaei et al., 2020; Zaw and Hlaing, 2024). Foundational STEM education scholarship has long recognized the educational potential of touch as a meaningful pathway for learning. Minogue and Jones (2006) described haptics as an underutilized sensory modality capable of enriching conceptual understanding through embodied interaction, while Jones et al. (2006a) demonstrated that tactile and haptic technologies enable learners to visualize and experience otherwise inaccessible microscopic scientific phenomena. For example, Richard et al. (1997) demonstrated how haptic interface kits could be used to teach dynamics and control concepts through active interaction, while Okamura et al. (2002) further emphasized the educational value of haptic systems for experiential learning and interactive problem solving.

Subsequent research expanded these applications to tactile models, immersive simulations, accessibility technologies, engineering education, multisensory environments (Dyzel et al., 2020; Jiang et al., 2025). Many of these approaches rely on underlying physical principles—such as vibration, pressure, frequency, and force response—that shape the sensory experiences generated through interaction (Kristiyanto et al., 2020). Understanding how these sensory signals are translated into meaningful learning experiences is important for clarifying the relationship between perception, embodied action and conceptual learning. Despite growing interest in tactile and haptic learning environments, research in this area remains fragmented across disciplines. Existing studies are distributed throughout diverse fields of inquiry and often emphasize technological innovation or accessibility features rather than explicitly examining how touch-based interaction functions as a mechanism of learning and cognition. Consequently, there remains limited integrative understanding of how tactile and haptic approaches translate physical phenomena into sensory experiences that support conceptual reasoning, participation, and engagement within STEM learning contexts (Crandall and Karadogan, 2021; Magaña and Balachandran, 2017; Minaker et al., 2016).

Scoping review methodology provides an appropriate approach for synthesizing research in emerging and interdisciplinary fields. Scoping reviews allow researchers to systematically map existing literature, identify patterns in intervention design, and clarify conceptual relationships across heterogeneous bodies of research (Arksey and O'Malley, 2005; Pham et al., 2014). Rather than evaluating effectiveness, scoping reviews focus on characterizing the range of approaches used within a field and identifying gaps that may guide future research.

The present study conducts a scoping review of tactile and haptic interaction in science learning settings. Rather than evaluating intervention effectiveness, the review maps how tactile and haptic pedagogical approaches are designed and how they translate physical phenomena into perceptible sensory experiences that support conceptual engagement. The synthesis examines the types of tactile and haptic interventions described in the literature, the sensory mechanisms through which these approaches operate, and the learning processes they support. Particular attention is given to how sensorimotor interaction allows learners to explore abstract scientific relationships through embodied and multisensory experience.

By synthesizing patterns across studies, this review clarifies how tactile and haptic learning environments link physical principles, sensory interaction, and pedagogical functions in ways that support conceptual reasoning and participation in STEM learning contexts. The analysis culminates in the Tactile and Haptic Interaction (THIn) conceptual model, which theoretically explains how embodied sensory interaction connects perception, action, and conceptual reasoning during learning. In doing so, the study contributes to educational psychology by identifying how multisensory interaction can function as a pathway for conceptual learning and by outlining directions for future research on tactile and haptic pedagogies.

## 2 Methodology

### 2.1 Review design

A scoping review methodology was employed to systematically map research on tactile and haptic interaction in STEM learning contexts and to examine how sensory interaction supports conceptual learning processes across diverse educational settings. Although tactile and haptic approaches are often discussed in relation to inclusive education and accessibility for deaf and hard-of-hearing (DHH) learners, the present review examines these approaches more broadly as learning mechanisms within STEM education. This perspective allows the synthesis to capture research across multiple disciplines—including science education, human–computer interaction, and perception science—where tactile interaction has been used to represent and explore scientific phenomena through embodied experience. Scoping reviews are appropriate for emerging and interdisciplinary areas where conceptual clarification and mapping of research activity are required rather than formal evaluation of intervention effectiveness (Marwah et al., 2025).

**Figure 1:**
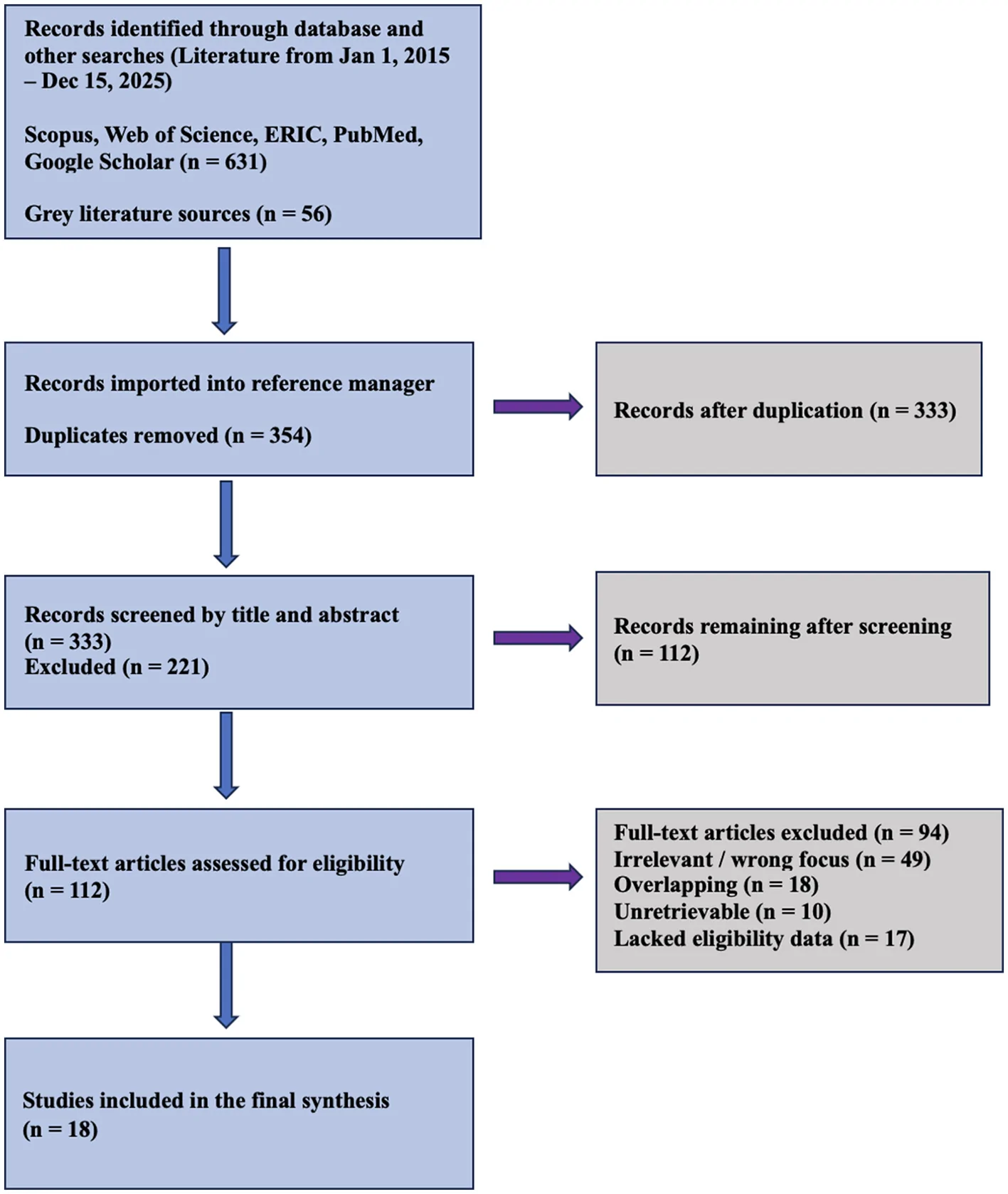
PRISMA flow diagram of study identification, screening, eligibility, and inclusion for tactile and haptic interaction in STEM learning.

The review followed the six-stage framework proposed by Arksey and O'Malley (2005) and later refined by Levac et al. (2010). Reporting adhered to the Preferred Reporting Items for Systematic Reviews and Meta-Analyses extension for Scoping Reviews (PRISMA-ScR) guidelines (Tricco et al., 2018). This approach enabled systematic identification, screening, and synthesis of literature examining tactile and haptic learning environments and the sensory mechanisms through which they support conceptual engagement with STEM phenomena. Figure 1 presents the PRISMA flow diagram of the primary study selection process.

### 2.2 Search strategy and timeframe

A systematic search strategy was implemented to identify literature examining tactile and haptic interaction in multisensory STEM learning environments, particularly studies addressing learning environments relevant to DHH learners or multisensory educational contexts more broadly. Searches were conducted across five major databases: Scopus, Web of Science, ERIC, PubMed, and Google Scholar.

The search covered publications from January 1, 2015 to December 15, 2025, with the final search conducted on December 15, 2025. This timeframe was selected to capture research reflecting the growing integration of tactile and haptic technologies within educational research and multisensory learning environments.

Search terms were organized into four conceptual clusters: (1) DHH learners or sensory accessibility, (2) tactile or haptic interaction, (3) STEM or physics learning, and (4) educational or instructional contexts. Boolean operators, truncation, and controlled vocabularies (e.g., MeSH and ERIC descriptors) were applied where appropriate. Search strings were adapted for each database. Database-specific search strategies and the number of records retrieved during the initial search are summarized in Table 1.

**Table 1:** Database-specific search strings and records retrieved during the primary search process.

Source	Fields searched	Search string (summary)	Hits (*n*)
Scopus	Title, Abstract, Keywords	(“deaf” OR “hard of hearing” OR “DHH”) AND (“tactile” OR “haptic” OR “touch-based”) AND (“STEM” OR “science” OR “physics”) AND (“instruction^*^” OR “pedagog^*^” OR “teaching” OR “learning” OR “instructional design” OR “applied learning” OR “STEM education”)	128
Web of Science (Core Collection)	Topic	TS=(“deaf” OR “hard of hearing” OR “DHH”) AND TS=(“tactile” OR “haptic” OR “touch-based”) AND TS=(“STEM” OR “science” OR “physics”) AND TS=(“instruction^*^” OR “pedagog^*^” OR “teaching” OR “learning” OR “instructional design” OR “applied learning” OR “STEM education”)	97
ERIC	Descriptors, Title, Abstract	(DE “Deafness” OR DE “Hearing Impairments” OR TI/AB “deaf” OR “hard of hearing” OR “DHH”) AND (TI/AB “tactile” OR “haptic”) AND (DE “STEM Education” OR DE “Science Education” OR TI/AB “STEM” OR “physics”) AND (DE “Teaching Methods” OR DE “Instructional Design” OR TI/AB “instruction^*^” OR “pedagog^*^” OR “skills based”)	103
PubMed	MeSH Terms; Title/Abstract	(“Deafness”[MeSH] OR “Hearing Loss”[MeSH] OR deaf OR “hard of hearing”) AND (tactile OR haptic) AND (STEM OR science OR physics) AND (education[MeSH] OR instruction^*^ OR pedagog^*^ OR skills based)	87
Google Scholar	Keyword search	Simplified keyword combinations; first 200 results screened per query	216
Subtotal—Databases	—	—	631
Gray literature	Platform-specific search	(deaf OR “hard of hearing”) AND (tactile OR haptic) AND (STEM OR physics) AND education	56
Total records identified	—	—	687

Table 1 documents records retrieved during the primary search process. To strengthen the comprehensiveness of the review and broaden coverage of mainstream STEM education literature, a supplementary targeted search and citation chaining process were conducted while preserving the original review timeframe and eligibility criteria. This supplementary process is presented separately in Supplementary Figure S1.

To strengthen the comprehensiveness of the review and broaden coverage of mainstream STEM education scholarship, a supplementary targeted search was conducted while preserving the original review timeframe, search boundaries, and eligibility criteria. Targeted searches were performed in the International Journal of Science Education (IJSE), Research in Science Education (RISE), Journal of Science Education and Technology (JSET), Science Education, and Computers and Education, complemented by backward and forward citation chaining of relevant studies. This process retrieved 22 additional records, resulting in the inclusion of five eligible studies and increasing the final synthesis from 18 to 23 studies and strengthening representation from mainstream STEM education literature. The supplementary search process is presented in Supplementary Figure S1.

### 2.3 Eligibility criteria

This scoping review prioritized studies examining tactile or haptic interaction within STEM learning environments relevant to DHH learners. In this review, tactile and haptic interventions refer to learning environments in which scientific phenomena are mediated through sensory interaction, including tactile models, force-feedback systems, vibrotactile interfaces, and other forms of touch-based representation.

Studies were included if they: (a) involved DHH learners or addressed learning environments designed for inclusive STEM education; (b) described a pedagogical activity, instructional tool, or learning environment rather than purely technical prototyping; and (c) provided sufficient detail to identify the sensory mechanisms involved (e.g., vibration, pressure, texture, or force feedback) and their role in supporting learning processes. Educational levels reported in the studies—including primary, secondary, tertiary, and vocational contexts—were considered when interpreting variations in instructional settings.

In addition to studies involving DHH learners, selected studies involving non-DHH populations were included when they provided detailed descriptions of tactile or haptic interaction mechanisms within STEM learning environments. These studies were retained to clarify the sensory–perceptual processes through which embodied interaction mediates conceptual engagement with scientific phenomena (Correia Lima et al., 2024; Wiebe et al., 2009). Because many DHH-focused educational studies emphasize accessibility or instructional implementation rather than the underlying sensory design of tactile learning tools, evidence from engineering, perception science, human–computer interaction, and mainstream STEM education literature provided important insights into how tactile signals—such as vibration, pressure, force feedback, and spatial structure—are generated, interpreted, and integrated into learning activities (Crandall and Karadogan, 2021; Dyzel et al., 2020; Jiang et al., 2025). Findings from non-DHH studies were interpreted only at the level of learning design and sensory interaction mechanisms and were not treated as evidence of learning outcomes for DHH learners. This analytical distinction allowed broader evidence on somatosensory interaction to be synthesized while ensuring that population-specific interpretations remained grounded in DHH-related studies.

Studies were excluded if they: (a) focused exclusively on clinical rehabilitation, audiological treatment, or medical device testing without an instructional STEM component; (b) addressed accessibility solely through visual or sign-language modalities without tactile or haptic interaction; (c) described tactile technologies at a purely technical or engineering level without learner engagement; or (d) lacked sufficient information to analyze the relationship between sensory interaction and learning processes. Only English-language publications were included.

Data were extracted on publication details, participant context, tactile or haptic intervention type, underlying sensory mechanisms, pedagogical purposes, and reported learning outcomes. The synthesis employed a descriptive and inductive thematic approach to identify patterns across studies, with particular attention given to how tactile interaction mediates learners' engagement with abstract scientific phenomena and how sensory feedback supports conceptual reasoning in STEM learning contexts.

Consistent with scoping review methodology, the review did not evaluate intervention effectiveness or compare learning outcomes across studies (Al-Hakeem et al., 2025; Pham Xuan, 2025). Instead, the analysis focused on mapping patterns in the design of tactile learning environments and identifying how sensory interaction contributes to conceptual engagement and participation in STEM learning activities. Table 2 summarizes the characteristics of the 23 studies included in the final synthesis.

**Table 2:** Characteristics of the 23 studies included in the final scoping review synthesis, organized according to their primary contribution to understanding tactile and haptic interaction in STEM learning.

References	Country/Region	Study design	Education level	STEM/Learning area	Participant characteristics (DHH where applicable)	Setting	Type of tactile, haptic, or embodied intervention
A. DHH and inclusive STEM education studies							
Utami et al. (2021)	Indonesia	Intervention/blended learning case study	Higher education	General education (critical thinking)	Deaf students in university	Higher education classroom (blended)	Structured blended learning model incorporating multimodal supports
David et al. (2023)	Multiple/global	Narrative review	Mixed	General education, ICT	Deaf and hard-of-hearing learners	School contexts	ICT and assistive tools including tactile interfaces
Constantinou et al. (2020)	Cyprus	Scoping review	Primary and secondary	General education/inclusion	Deaf students in mainstream schools	Mainstream classrooms	Technology supports including tactile and visual-haptic tools
Abdul Rahim et al. (2024)	Malaysia	Descriptive study	Tertiary (polytechnic)	Technology education	Deaf and hard-of-hearing students	Polytechnic classrooms	Educational technology use including tactile resources
Chelladurai et al. (2024)	USA	Design and usability study	Mixed	Accessibility/sound perception	Deaf and hard-of-hearing users	Virtual reality environments	SoundHapticVR spatial vibrotactile system
Dostal et al. (2025)	USA	Scoping review	School-age	Literacy/learning	Deaf students	School settings	Signed-language and multimodal literacy interventions
B. Mainstream STEM education literature							
Jones et al. (2006a)	USA	Exploratory intervention study	Secondary	Biology (cell structure)	Students with visual impairment	Science classroom	Tactile exploration of microscopic cell structures
Jones et al. (2006b)	USA	Experimental study	Secondary	Science education	Secondary students	Science classroom	Haptic augmentation integrated into science instruction
Wiebe et al. (2009)	USA	Experimental study	Secondary	Physics (levers and mechanics)	Secondary students	Classroom/simulation	Haptic feedback within lever simulations
Lindgren et al. (2016)	USA	Mixed-methods intervention	Primary	General STEM	Primary learners (inclusive context)	Classroom and mixed-reality space	Embodied mixed-reality simulations
Jones et al. (2016)	USA	Experimental intervention	Secondary	Physics (thermal energy, pressure, motion)	Secondary learners	Classroom/simulation	Visuohaptic simulations for scientific concepts
Magaña and Balachandran (2017)	USA	Experimental study	Tertiary	Engineering education	Undergraduate students	University classroom	Haptic-supported representational learning activities
Neri et al. (2018)	Italy	Experimental study	Secondary	Physics (friction)	Secondary students	Classroom/simulation	Visuo-haptic simulations for friction concepts
Walsh and Magana (2023)	USA	Experimental study	Tertiary	Engineering/statics	General learners	University classroom/laboratory	Physical manipulatives and visuohaptic simulations
Johnson-Glenberg et al. (2023)	USA	Randomized controlled study	Tertiary	Chemistry (titration)	University students	University laboratory	Mixed-reality environments with passive haptics
C. Engineering, haptics, perception science, and broader multisensory learning literature							
Cuppone et al. (2016)	Italy	Experimental laboratory study	Mixed	Somatosensory/motor learning	Not DHH-specific	Laboratory	Proprioceptive robot training with vibrotactile feedback
Martinez et al. (2016)	USA	Prototype and pilot study	Secondary/tertiary	Engineering/technology	Students in demonstration contexts	Educational demonstrations/laboratory	Three-dimensional printed haptic instructional devices
MacLean et al. (2017)	Canada	Conceptual synthesis	Mixed	Haptics/interaction design	Not DHH-specific	Not specific	Multisensory haptic design principles
Shaikh et al. (2017)	USA	Mixed-methods study	Tertiary	Engineering/technology learning	Undergraduate students	University classroom	Haptic-enabled interactive learning experience
Lelevé et al. (2020)	International	Conceptual and design demonstration	Vocational/training	Technical training	Not DHH-specific	Simulation environments	Haptic training simulators
Sanfilippo et al. (2022)	Europe	System development and pilot study	Higher education e-learning	General STEM	Not DHH-specific	Online learning	Multimodal auditory-visual-tactile learning framework
See et al. (2022)	Global	Review	Mixed	Tactile and haptic perception science	Not DHH-specific	Not specific	Mechanisms of touch, texture, and haptic sensing
Mutis and Oberemok (2025)	USA	Conceptual framework	Higher education (engineering)	Engineering learning	Not DHH-specific	Higher education contexts	Haptic interaction conceptual framework

Studies are organized according to their primary contribution to the review rather than publication year. While the review prioritized STEM learning environments relevant to deaf and hard-of-hearing (DHH) learners, selected studies involving non-DHH populations were intentionally retained when they provided detailed descriptions of tactile and haptic sensory mechanisms, embodied interaction processes, and learning design features that are often underrepresented in DHH-specific educational research. These studies were interpreted at the level of sensory interaction design rather than as evidence of learning outcomes for DHH learners.

## 3 Results

### 3.1 Forms of tactile and haptic interaction in STEM learning environments

Across the included studies, tactile and haptic learning environments were used to support conceptual engagement with STEM phenomena by translating information typically conveyed through auditory or abstract visual representations into perceptible tactile experiences. Rather than functioning solely as assistive technologies, these environments created opportunities for learners to explore scientific concepts through direct sensorimotor interaction (Chelladurai et al., 2024; Jones et al., 2016; Johnson-Glenberg et al., 2023; Lindgren et al., 2016).

**Figure 2:**
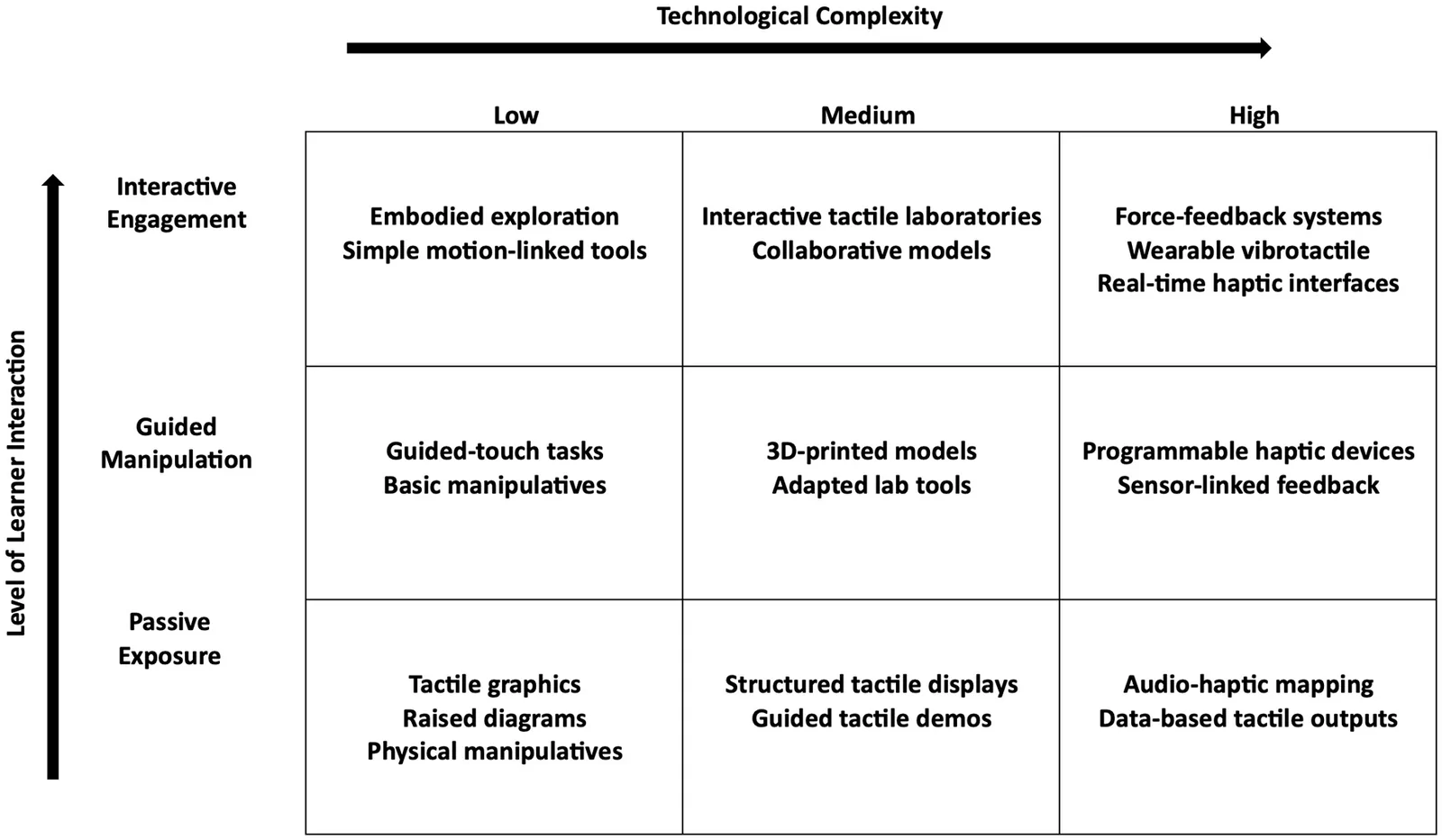
Typology of tactile and haptic pedagogical interventions used in STEM education for DHH learners. The horizontal axis represents the technological complexity, while the vertical axis represents the level of learner interaction.

Consistent with the goals of a scoping review, the literature was mapped to characterize the range of tactile learning environments and interaction patterns rather than to evaluate comparative effectiveness. Through cross-study comparison of intervention characteristics, tactile and haptic learning environments were organized into a two-dimensional typology based on technological complexity and level of learner interaction. Figure 2 presents the typology of tactile and haptic learning environments identified in the reviewed studies, illustrating how differences in technological complexity correspond to varying levels of learner interaction and sensorimotor engagement.

Low-technology learning environments included tactile graphics, raised diagrams, and physical manipulatives that supported spatial reasoning and structural comparison. These environments allowed learners to explore spatial relationships and system structures through guided touch (Lindgren et al., 2016; Martinez et al., 2016; Walsh and Magana, 2023).

Mid-level environments incorporated three-dimensional models and adapted laboratory tools that conveyed motion, gradients, or structural change through tactile interaction. These tools often supported inquiry-based activities in which learners manipulated representations to investigate relationships between variables (Johnson-Glenberg et al., 2023; Lindgren et al., 2016; Walsh and Magana, 2023).

High-technology environments included vibrotactile systems, wearable devices, force-feedback interfaces, and interactive simulations that translated data or sound into patterned tactile signals. These systems enabled real-time exploration of dynamic processes such as wave propagation, motion, or system dynamics (Johnson-Glenberg et al., 2023; MacLean et al., 2017; Mutis and Oberemok, 2025).

The reviewed literature indicates that technological complexity did not correspond directly to stronger learning outcomes. Instead, differences in technological form reflected varying instructional purposes, resource constraints, and pedagogical strategies. Low-technology tools were commonly used in classroom-based conceptual exploration, while higher-technology environments were more frequently associated with interactive or simulation-based inquiry activities. Overall, the reviewed literature suggests that the effectiveness of tactile learning environments depends less on technological sophistication than on how sensory interaction is integrated into learning activities and instructional scaffolding (Jones et al., 2016; Sanfilippo et al., 2022; Shaikh et al., 2017; Walsh and Magana, 2023). Studies across mainstream STEM education literature further highlighted the importance of embodied interaction, representational competence, and force-mediated conceptualization in supporting STEM learning. These studies demonstrated that tactile engagement enables learners to develop mental models of scientific phenomena through active exploration rather than passive observation (Jones et al., 2016; Magaña and Balachandran, 2017; Neri et al., 2018; Wiebe et al., 2009).

### 3.2 Sensory–perceptual mechanisms supporting conceptual learning

The reviewed studies demonstrated that tactile and haptic learning environments rely on identifiable sensory mechanisms that allow learners to perceive and interpret scientific relationships through bodily interaction. These mechanisms translate physical properties such as force, vibration, pressure, motion, and spatial structure into perceptible tactile signals (MacLean et al., 2017; Mutis and Oberemok, 2025; See et al., 2022). Through these sensory mappings, abstract STEM phenomena—such as energy transfer, equilibrium, system dynamics, or wave behavior—can be experienced through direct interaction rather than symbolic representation alone (Walsh and Magana, 2023).

From a perceptual perspective, these environments engage multiple components of the somatosensory system, including cutaneous touch, proprioception, and kinesthetic awareness. Sensorimotor interaction enables learners to perceive gradients, resistance, motion, structural boundaries, and temporal change through exploration of objects or interfaces (Cuppone et al., 2016; Johnson-Glenberg et al., 2023; See et al., 2022). Table 3 presents the relationships among physical principles, sensory mechanisms, and pedagogical functions identified in the literature, illustrating how different tactile signals translate physical phenomena into interpretable learning experiences. Three recurring patterns of sensory mediation were identified.

**Table 3:** Synthesized relationships between physical principles, somatosensory mechanisms, and pedagogical functions in tactile and haptic STEM learning environments.

Physical principle	Somatosensory mechanism engaged	Example intervention	Supported learning function	Representative citations
Vibration (frequency, amplitude)	Cutaneous touch; temporal pattern perception	Vibrotactile wristbands or pads translating sound or data into pulses	Representing wave behavior, periodicity, and signal change	Walsh and Magana, 2023; Jones et al. (2006b)
Force and resistance	Proprioception; kinesthetic feedback from muscles and joints	Force-feedback controllers simulating push–pull or resistance	Experiencing forces, acceleration, and system constraints	Cuppone et al., 2016; Wiebe et al., 2009
Pressure and load	Tactile pressure receptors; graded force sensing	Pressure-responsive surfaces or pads	Understanding gradients, equilibrium, and comparative magnitudes	See et al., 2022
Motion and displacement	Kinesthesia; spatial awareness	Movable tactile models and manipulatives	Tracking trajectories, relative movement, and positional change	Lindgren et al., 2016; Neri et al., 2018
Texture and contour	Fine tactile discrimination (fingertips)	Raised diagrams, layered surfaces, and textured maps	Distinguishing structure, hierarchy, boundaries, and transitions	MacLean et al., 2017; Jones et al. (2006a)
Spatial layout/topology	Whole-hand exploration; multisite tactile integration	Three-dimensional printed models and physical replicas	Building mental models of complex systems and spatial relations	Constantinou et al., 2020; Magaña and Balachandran, 2017
Temporal patterning	Integration of repeated rhythmic cues over time	Wearable haptics delivering patterned alerts	Sequencing steps, detecting change, and monitoring processes	Martinez et al., 2016; Jones et al. (2006b)

Representative citations are illustrative rather than exhaustive. The relationships presented were synthesized from the overall evidence base of 23 included studies. Studies primarily addressing broader pedagogical, accessibility, review, or theoretical considerations informed the synthesis but were not directly mapped to specific physical principles.

First, vibrotactile representations translated dynamic or oscillatory phenomena—such as wave propagation, signal patterns, or frequency change—into patterned vibration that could be perceived through the skin. These representations allowed learners to experience temporal variation and periodicity through tactile cues rather than visual or auditory signals (Chelladurai et al., 2024; Walsh and Magana, 2023).

Second, force-feedback and resistance-based interactions enabled learners to perceive mechanical relationships such as equilibrium, constraint, or applied force through kinesthetic feedback during manipulation of physical or virtual systems. In these environments, conceptual understanding emerged through the perception of resistance, balance, or motion constraints experienced during interaction and through learners' ability to infer force relationships and system behavior via embodied manipulation (Cuppone et al., 2016; Magaña and Balachandran, 2017; Wiebe et al., 2009).

Third, spatial–structural tactile representations, including raised diagrams, textured surfaces, and three-dimensional models, supported exploration of spatial relationships and system topology through distributed tactile exploration (Johnson-Glenberg et al., 2023; Walsh and Magana, 2023).

Across these categories, a consistent design logic becomes visible: physical variables are translated into tactile signals that correspond to interpretable conceptual features of scientific phenomena. Through this mapping, sensorimotor interaction functions as a perceptual bridge between physical processes and conceptual reasoning. Rather than serving solely as accessibility adaptations, these sensory mappings allow learners to interpret relationships between variables through embodied exploration and sensorimotor feedback (MacLean et al., 2017; Mutis and Oberemok, 2025; Walsh and Magana, 2023).

However, the articulation of these sensory–conceptual mappings was inconsistent in the literature. In many cases, interventions were described primarily in terms of device functionality rather than the perceptual mechanisms through which learning occurred. As a result, the relationship between tactile signals—such as amplitude, frequency, resistance, or spatial structure—and targeted conceptual outcomes was often only implicitly described. This pattern highlights the need for clearer reporting of sensory–cognitive design principles in future research on tactile learning environments (MacLean et al., 2017; Mutis and Oberemok, 2025).

### 3.3 Cross-study themes in tactile and haptic learning processes

The synthesis generated themes through cross-study analysis of intervention design, sensory mechanisms, and reported learning outcomes across the 23 included studies. Rather than representing participant-derived narratives, these themes reflect recurring patterns in how tactile interaction was used to support conceptual learning.

#### 3.3.1 Theme 1: translating physical phenomena into tactile representations

Across studies, tactile and haptic learning environments consistently translated physical principles—such as motion, force, vibration, or wave behavior—into perceptible tactile signals. These representations allowed learners to explore otherwise invisible or abstract processes through guided interaction and sensory feedback (See et al., 2022). Through tactile exploration, learners were able to interpret relationships between variables and construct mental models of system behavior (Chelladurai et al., 2024; Johnson-Glenberg et al., 2023; Walsh and Magana, 2023). This process of conceptualization was further reinforced by studies demonstrating that tactile interaction supports learners in inferring force–distance relationships, spatial configurations, and dynamic system behaviors through embodied exploration (Magaña and Balachandran, 2017; Wiebe et al., 2009).

#### 3.3.2 Theme 2: embodied exploration as a pathway for conceptual reasoning

Learners were commonly positioned as active participants who explored and manipulated tactile representations through embodied action. Touch-based interaction enabled learners to test ideas, examine relationships, and reason through problems in ways that complemented visual or symbolic representations (Johnson-Glenberg et al., 2023; Walsh and Magana, 2023). Evidence from mainstream STEM education studies emphasized the role of tactile interaction in developing representational competence, enabling learners to infer force relationships, spatial structures, and system behaviors through sensorimotor experiences (Magaña and Balachandran, 2017; Neri et al., 2018; Wiebe et al., 2009). In this sense, touch-based interaction functioned as an epistemic resource that supported inquiry-based reasoning rather than merely serving as an accessibility adaptation (Lindgren et al., 2016).

#### 3.3.3 Theme 3: tactile interaction as scaffolding for procedural engagement

Several studies described tactile learning environments as providing structured guidance that supported procedural engagement with scientific activities. Through real-time tactile feedback, learners were able to practice sequences of actions, monitor system responses, and refine their interactions during experimentation. These forms of scaffolding supported procedural reasoning and helped learners coordinate perception and action when engaging with scientific tasks (Johnson-Glenberg et al., 2023; Shaikh et al., 2017; Utami et al., 2021).

### 3.4 Tactile and haptic interaction (THIn) model in STEM conceptual learning

Synthesizing patterns across the reviewed studies, this analysis proposes the Tactile and Haptic Interaction (THIn) conceptual model, explaining how tactile learning environments translate physical phenomena into sensory interaction and pedagogical processes that support conceptual understanding in STEM contexts. The model illustrates a sequence of relationships connecting four elements: (1) physical phenomena represented in learning environments, (2) sensory interaction mechanisms such as vibration, pressure, or force feedback, (3) pedagogical functions that structure exploration and feedback, and (4) learning processes related to conceptual understanding and procedural engagement. Within this framework, represented in the THIn conceptual model, tactile interaction operates as a sensory–cognitive pathway linking perception, action, and conceptual reasoning during embodied exploration.

**Figure 3:**
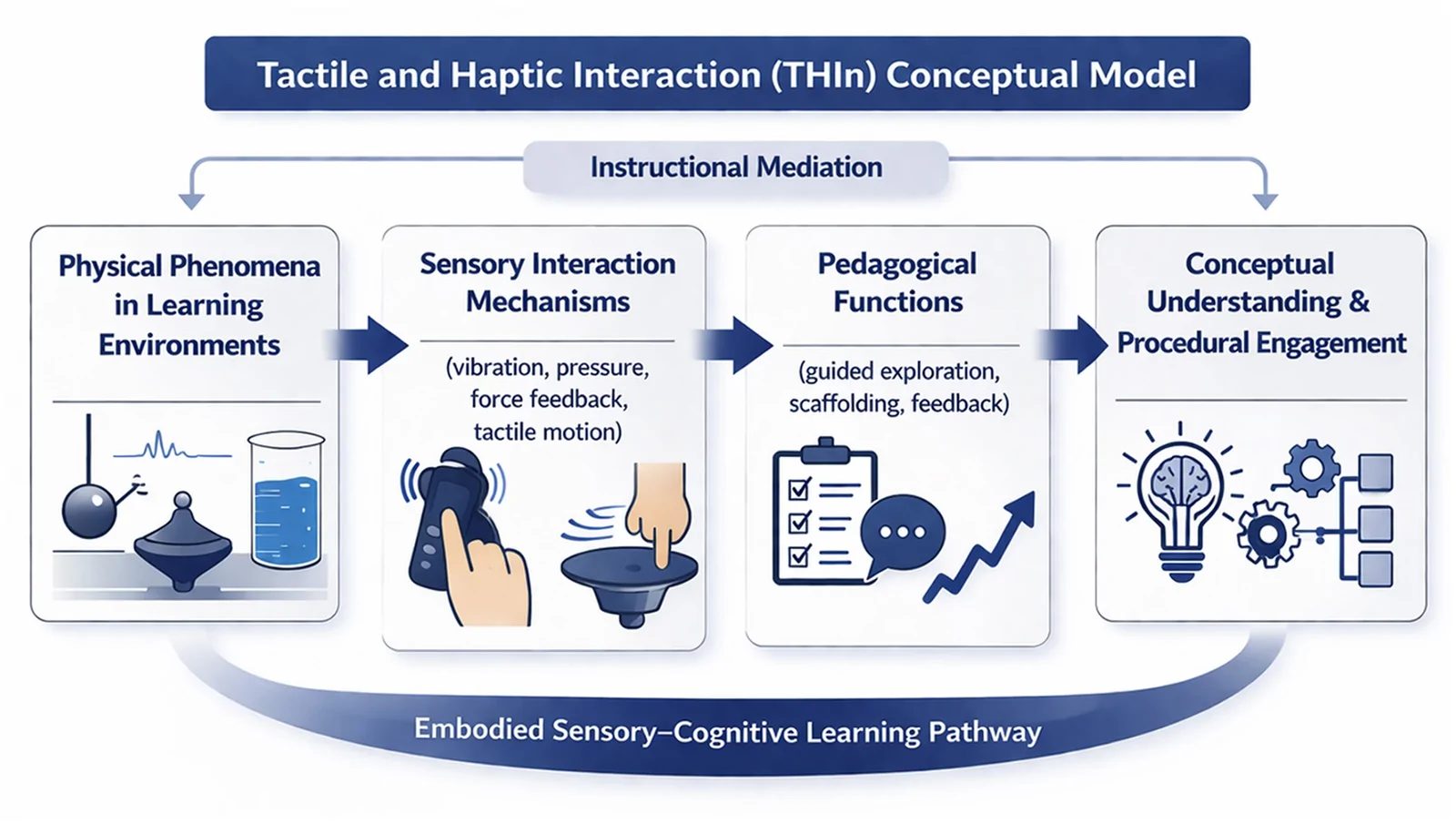
Tactile and Haptic Interaction (THIn) conceptual model illustrating how physical principles are translated into sensory interaction mechanisms and pedagogical functions that support conceptual learning through embodied interaction in STEM environments.

Taken together, these findings converge in the THIn conceptual model (Figure 3), which illustrates how physics-informed tactile and haptic designs translate physical principles into sensory mechanisms and pedagogical functions. These cross-disciplinary sensory–representational frameworks illustrate how technical design choices in tactile learning environments structure learning experiences through embodied interaction (MacLean et al., 2017; Mutis and Oberemok, 2025; Walsh and Magana, 2023). In the model, instructional mediation represents the role of pedagogical design and teacher guidance in translating sensory interaction into meaningful conceptual learning.

Learning through touch begins with the translation of physical principles—such as vibration, force, pressure, motion, and texture—into tangible representations. These representations are mediated through sensory mechanisms including vibrotactile feedback, force-feedback interfaces, and active manipulation. Through these mechanisms, haptic interaction provides perceptual cues that structure pedagogical functions such as representing otherwise invisible phenomena across STEM domains, guiding exploratory action, simulating experimental conditions, and providing feedback during learning activities (Chelladurai et al., 2024; Cuppone et al., 2016; Johnson-Glenberg et al., 2023).

These functions support conceptual understanding, procedural engagement, and broader participation in STEM learning by enabling learners to interpret scientific relationships through tactile and kinesthetic feedback. In aggregate, these learning processes are conceptually associated with procedural competencies such as precision handling, sequencing, troubleshooting, and safety awareness that resemble task structures encountered in laboratory or technical learning activities.

Analysis of the reviewed studies indicates stronger evidence for conceptual understanding and procedural engagement than for validated transfer of skills beyond the learning environment. While many interventions describe task-relevant behaviors such as sequencing or precision handling, relatively few studies provide longitudinal or assessment-based evidence demonstrating sustained skill development. This pattern highlights a structural imbalance between technological innovation and systematic evaluation within the field (Lelevé et al., 2020; Mutis and Oberemok, 2025).

Table 4 synthesizes the learning and skill-related domains reported across the included studies, linking observed learning affordances with interpretive analysis of how tactile and haptic interaction may support conceptual and procedural engagement in STEM learning contexts. These domains are interpreted at the level of conceptual affordances rather than demonstrated vocational or employment outcomes, with representative evidence drawn from the final set of 23 studies (Utami et al., 2021).

**Table 4:** Synthesized learning affordances and skill-related outcomes associated with tactile and haptic STEM learning environments.

Learning affordance or skill-aligned domain	Conceptual evidence reported across studies	Interpretive synthesis (scoping review analysis)	Representative citations
Conceptual access to abstract STEM phenomena	Tactile and haptic representations enabled learners to engage with phenomena such as waves, motion, gradients, and system behavior through embodied interaction and sensory feedback.	May plausibly support conceptual understanding by providing non-auditory representational pathways aligned with abstract reasoning.	Jones et al., 2016; Walsh and Magana, 2023; Johnson-Glenberg et al., 2023; Neri et al., 2018; Magaña and Balachandran, 2017
Participation and epistemic inclusion	Learning environments incorporating touch and embodied interaction reported higher engagement, confidence, and learner participation in inquiry activities.	May scaffold inclusive participation by reducing reliance on auditory channels and expanding epistemic access.	Dostal et al., 2025; Constantinou et al., 2020; David et al., 2023; Jones et al. (2006a)
Procedural engagement and task sequencing	Multi-step activities supported by tactile cues, resistance feedback, or embodied manipulation enabled learners to follow and adjust procedural sequences.	May support preparatory procedural fluency by aligning sensory cues with task structure and sequencing.	Martinez et al., 2016; Johnson-Glenberg et al., 2023; Shaikh et al., 2017; Wiebe et al., 2009
Precision handling and force regulation	Force-feedback and resistance-based systems allowed learners to experience controlled force application, constraint, and adjustment.	May plausibly scaffold precision handling skills relevant to technical and laboratory practices without implying workplace transfer.	Cuppone et al., 2016; Mutis and Oberemok, 2025; Wiebe et al., 2009
Safety awareness and error monitoring	Haptic systems provided corrective feedback and constraint cues during simulated task execution.	May support early safety awareness and error detection through guided rehearsal in low-risk environments.	Lelevé et al., 2020; Johnson-Glenberg et al., 2023
Troubleshooting and adaptive adjustment	Interactive tactile systems enabled learners to detect variation, respond to feedback, and modify actions during task performance.	May scaffold adaptive problem-solving and troubleshooting behaviors aligned with technical task contexts.	Martinez et al., 2016; Shaikh et al., 2017; Wiebe et al., 2009
Technology–livelihood orientation	Simulated or embodied learning environments resembled elements of technical or vocational activity (e.g., tool handling, process control, and monitoring).	Conceptually aligned with preparatory skill scaffolding for technology-oriented learning contexts without implying employability or labor-market outcomes.	Utami et al., 2021; Abdul Rahim et al., 2024; Sanfilippo et al., 2022
Inclusive and accessible learning design	Tactile and haptic supports were positioned as part of broader inclusive or assistive learning ecosystems for deaf and hard-of-hearing learners.	May inform inclusive instructional design by illustrating how tactile interaction can function alongside visual and signed modalities.	Chelladurai et al., 2024; Constantinou et al., 2020; David et al., 2023; Jones et al. (2006a)

Representative citations are illustrative rather than exhaustive. The synthesized learning affordances were informed by the overall evidence base of 23 included studies. Studies primarily addressing broader theoretical, review-based, or contextual perspectives informed the synthesis without necessarily contributing to every learning domain.

## 4 Discussion

This scoping review synthesized evidence from 23 studies examining tactile and haptic interaction in STEM learning environments in order to clarify how sensory interaction contributes to conceptual learning. The reviewed studies indicate that tactile and haptic pedagogies consistently translate physical phenomena—such as vibration, force, pressure, motion, and spatial structure—into perceivable sensory signals that learners could explore through embodied interaction. The body of evidence reveals a recurring design pattern in which physical principles are encoded into tactile representations, structured through pedagogical activities, and interpreted through sensorimotor exploration. This pattern aligns with embodied and multisensory learning perspectives, which suggest that conceptual understanding emerges when learners actively coordinate perception, movement, and interaction rather than passively receive information (Johnson-Glenberg et al., 2023; Lindgren et al., 2016; Walsh and Magana, 2023). These studies suggest that tactile and haptic pedagogies function most effectively as integrated representational systems rather than isolated technological tools. This synthesis broadens the conceptual evidence base by demonstrating that tactile and haptic learning environments support conceptual understanding through embodied interaction across diverse STEM contexts, extending beyond accessibility-focused applications alone. This broader perspective is further supported by visuohaptic STEM studies, with Sanchez (2013) and Neri et al. (2018) demonstrating that coordinated visual and tactile interaction can strengthen understanding of complex STEM concepts by allowing abstract physical relationships to be explored through multisensory experiences.

Within this framework, and as represented in the Tactile and Haptic Interaction (THIn) conceptual model, tactile interaction functions as a sensory–cognitive pathway linking perception, action, and conceptual reasoning during learning. This conceptual linkage clarifies the embodied mechanisms through which multisensory interaction can support conceptual understanding in STEM contexts. Rather than operating solely as accessibility adaptations, tactile and haptic approaches function as multisensory representational systems that allow learners to engage with otherwise abstract or nonobservable scientific relationships. At the same time, the reviewed studies provide stronger evidence for conceptual engagement and procedural interaction than for validated transfer of skills beyond the learning environment, highlighting the need for further research examining how tactile learning designs support sustained conceptual development across educational contexts. These relationships are synthesized in the THIn conceptual pathway model, which illustrates how tactile learning environments link physical principles, sensory mechanisms, pedagogical functions, and learning processes within embodied STEM learning experiences.

### 4.1 Implications for inclusive pedagogy

These findings reposition tactile and haptic pedagogies as epistemic resources within inclusive STEM education rather than as compensatory or assistive add-ons. By enabling learners to engage with abstract concepts through embodied interaction, tactile and haptic designs expand how knowledge can be represented, explored, and negotiated in the classroom. Inclusive pedagogy, in this sense, emerges not from specific technologies but from the alignment of sensory experience, pedagogical function, and learning goals (Rončević and Rieckmann, 2025; Sanger, 2020).

The positioning of tactile and haptic systems as epistemic resources is reflected across multiple included studies. For example, Walsh and Magana (2023), Lindgren et al. (2016), and Johnson-Glenberg et al. (2023) demonstrate how embodied interaction supports conceptual reasoning beyond compensatory access. Similarly, Utami et al. (2021) and Dostal et al. (2025) illustrate how tactile models function as representational tools that restructure participation rather than merely accommodate learning differences.

**Figure 4:**
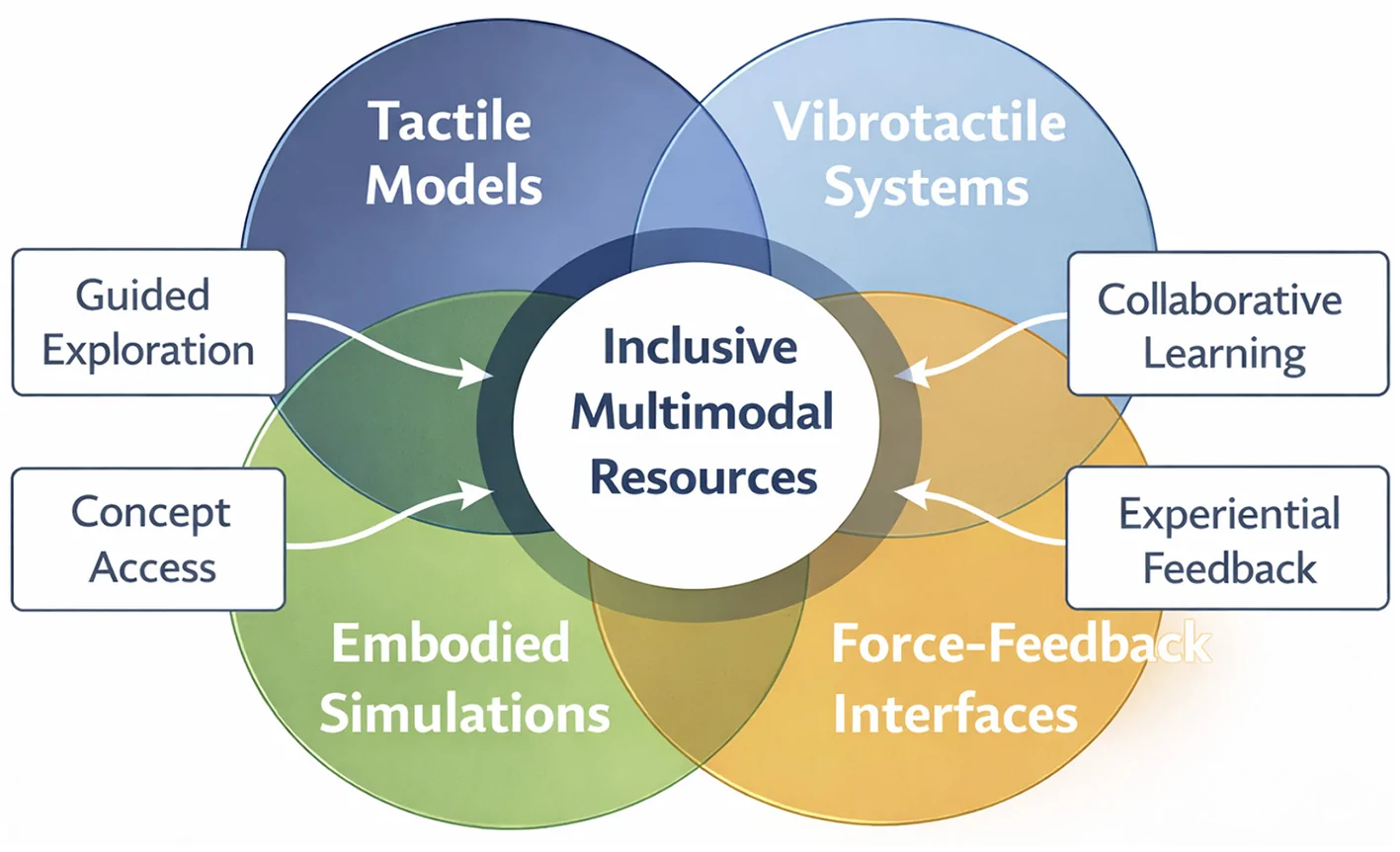
Overlapping instructional functions of tactile and haptic modalities in inclusive STEM learning environments. The diagram illustrates how different tactile and haptic technologies can support shared pedagogical processes—such as guided exploration, conceptual access, collaborative learning, and experiential feedback—functioning as inclusive multimodal resources for both deaf and hard-of-hearing and hearing learners.

Figure 4 highlights the overlapping instructional functions of different tactile and haptic modalities, illustrating how multiple technological forms can support similar pedagogical processes within inclusive STEM classrooms. This demonstrates that inclusive impact depends on pedagogical alignment rather than technological sophistication alone. This perspective also extends to inclusive classroom contexts in which DHH learners participate together with hearing peers. In such settings, tactile and haptic representations can function as shared multimodal resources that support joint engagement and understanding across sensory differences. Evidence from Utami et al. (2021), David et al. (2023), and Constantinou et al. (2020) further suggests that inclusive participation is strengthened when multiple sensory channels are intentionally integrated into instructional design. Together, these studies reinforce the view that inclusion is achieved not through technology alone but through pedagogical structures that enable shared meaning-making among diverse learners.

### 4.2 Conceptual alignment with technical learning contexts

Tactile and haptic learning environments occasionally resemble aspects of technical or laboratory activity because they require learners to coordinate perception and action through tactile feedback. Within the conceptual pathway model, these interactions can approximate task structures found in technical learning contexts, such as manipulating tools, monitoring system responses, or adjusting actions based on feedback. Several included studies describe learning environments in which tactile interaction supported procedural engagement with simulated or experimental activities (Martinez et al., 2016; Mutis and Oberemok, 2025; Shaikh et al., 2017). However, consistent with previous research, empirical evidence demonstrating transfer of these learning experiences to formal vocational certification or workplace performance remains limited (Abdul Rahim et al., 2024; Chelladurai et al., 2024).

Importantly, the review also reveals a persistent translational gap: few studies examine whether learning achieved through tactile or haptic environments transfers to workplace performance, formal vocational assessment, or certification pathways. As a result, technology–livelihood connections identified in this review must be interpreted as conceptual and proxy-based rather than outcome-based. Addressing this gap will require closer alignment with vocational curricula, industry standards, and local labor contexts in future research examining how tactile learning environments support participation in technical learning pathways. This observation is consistent with Lelevé et al. (2020) and Mutis and Oberemok (2025), which highlight the potential of tactile and haptic systems in technical training environments while simultaneously noting the limited evidence regarding long-term transfer to authentic workplace performance and competency development.

### 4.3 Sustainability and scalability

**Figure 5:**
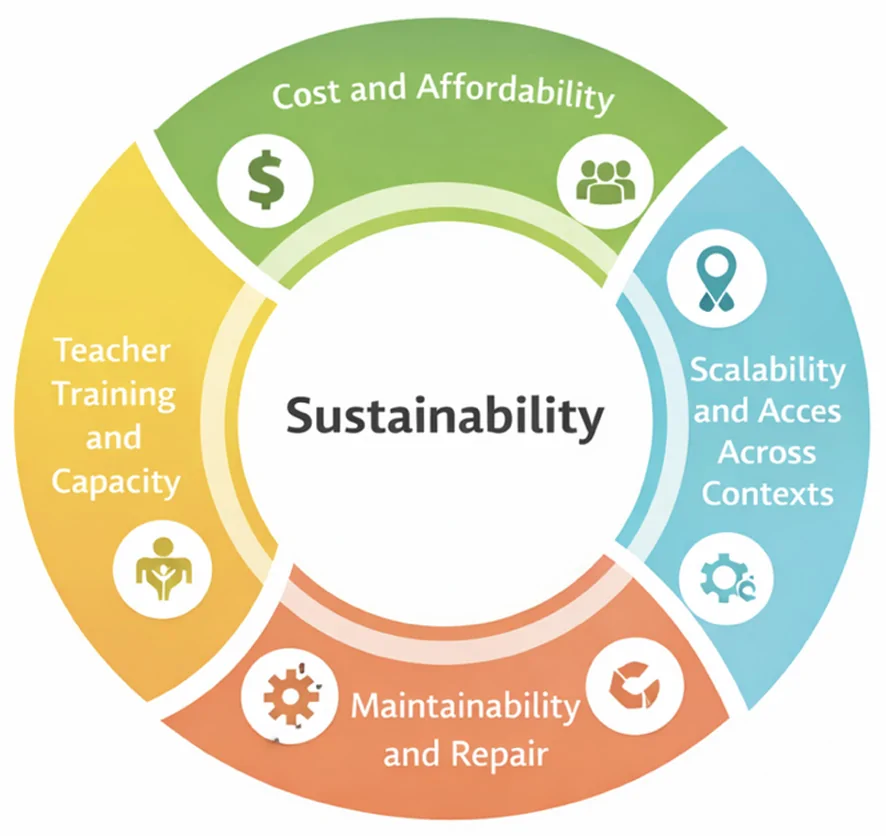
Sustainability framework for tactile and haptic pedagogies within Education for Sustainable Development (ESD). The framework illustrates four system-level conditions that influence sustainable implementation of tactile and haptic learning environments: cost and affordability, scalability across educational contexts, maintainability and repair, and teacher training and capacity. Together, these factors emphasize that long-term sustainability depends on pedagogical integration and institutional readiness rather than on technological complexity alone.

As illustrated in Figure 5, sustainability and scalability within the scope of ESD depend less on technology type than on system-level conditions—including cost and affordability, maintainability and repair, teacher training and capacity, and scalability across diverse educational contexts (Sihombing et al., 2024; Syifa et al., 2024). The figure emphasizes that long-term implementation depends more on contextual integration and teacher mediation than on device complexity. High-technology systems such as those described by MacLean et al. (2017) and See et al. (2022) offer sophisticated sensory feedback. In contrast, lower-cost and classroom-integrated approaches reported by Jones et al. (2016) and (Sanfilippo et al., 2022) suggest that sustainability depends less on device complexity and more on pedagogical alignment and teacher mediation, a pattern also noted by Lelevé et al. (2020). Sanfilippo et al. (2022) likewise emphasized that sustainable multisensory STEM environments depend on thoughtful integration of immersive technologies with pedagogically meaningful haptic experiences rather than technology adoption alone.

Scalable implementation depends on instructional practices that preserve pedagogical meaning over time. Evidence from structured inquiry and project-based physics instruction similarly demonstrates that sustained conceptual learning depends on pedagogical scaffolding and guided exploration rather than on instructional tools alone (Salibay, 2026). The review indicates that sensory input from tactile or haptic devices alone is insufficient for durable learning without intentional teacher mediation. Educators play a central role in translating physical sensations (e.g., vibration or resistance) into conceptual understanding by synchronizing tactile experiences with visual representations, gestures, and sign language where appropriate. Sustained implementation hinges on professional preparation that enables teachers to facilitate “minds-on” reflection from “hands-on” interaction, ensuring that tactile experiences are integrated into coherent learning trajectories rather than used as stand-alone activities. Moreover, Utami et al. (2021) and Sanfilippo et al. (2022) demonstrate that sustainable implementation depends not only on technological availability but also on educators' capacity to integrate multimodal supports within coherent instructional sequences. This finding reinforces the importance of teacher preparedness as a central determinant of long-term implementation success.

This synthesis clarifies how physical principles, sensory mechanisms, and pedagogical functions interact within tactile and haptic learning environments in inclusive STEM education. By making these relationships explicit, the study offers a conceptual and reporting framework that future intervention studies can operationalize and evaluate.

### 4.4 Limitations and directions for future research

This review has several limitations related to the composition of the evidence base. Only a minority of the included studies directly involved deaf or hard-of-hearing (DHH) learners, while most examined tactile or haptic interaction in broader STEM learning contexts. These studies were intentionally retained because they provided detailed descriptions of how physical principles are translated into sensory mechanisms and pedagogical functions—information that is often insufficiently articulated in DHH-specific educational research. Evidence from non-DHH studies was interpreted strictly at the level of design principles and sensory–pedagogical mechanisms rather than as empirical evidence of learning outcomes for DHH learners.

This limitation highlights an important direction for future research. Empirical studies that integrate explicit reporting of sensory–physical design logic with rigorous evaluation of conceptual learning outcomes are needed in DHH STEM education contexts. Future work should also examine how tactile and haptic learning environments support conceptual development across different educational levels and learning settings. Such work would strengthen the evidence base by connecting physics-informed design principles with population-specific learning outcomes in DHH STEM education. Adopting evaluative approaches used in visuohaptic STEM research may further strengthen future investigations. For example, Wiebe et al. (2009), Sanchez (2013), and Neri et al. (2018) demonstrated how simulated touch environments can be systematically evaluated to examine conceptual understanding across different STEM domains.

### 4.5 Managerial and policy implications

Extending the implications of this review beyond classroom practice, realizing the educational potential of tactile and haptic pedagogies for deaf and hard-of-hearing learners requires coordinated institutional and policy support. Rather than treating tactile resources solely as accommodations, curricular initiatives may consider integrating multimodal and physics-informed instructional materials within mainstream STEM learning environments. At the same time, sustained professional development is essential so that teachers can effectively combine tactile interaction with visual representations, gesture, and sign language during instruction. This recommendation is consistent with findings reported by David et al. (2023), Abdul Rahim et al. (2024), and Constantinou et al. (2020), which collectively emphasize that institutional readiness, teacher preparedness, and sustained access to multimodal resources are critical factors for successful implementation across educational contexts.

Institutional leaders may also consider procurement strategies that balance affordability and durability by combining low-cost tactile resources with selectively deployed high-technology systems. Such approaches may help sustain implementation in resource-constrained educational contexts. Partnerships between schools, technical training institutions, and industry stakeholders may also support the alignment of classroom learning experiences with broader STEM skill development pathways. Finally, policy initiatives that encourage local fabrication of tactile learning materials—such as 3D-printed resources—may reduce costs while supporting inclusive innovation and community-based design capacity (David et al., 2023; UNESCO, 2025).

## 5 Conclusion

Tactile and haptic pedagogies offer promising pathways for expanding multisensory engagement in STEM learning environments, with particular relevance to deaf and hard-of-hearing (DHH) learners. This scoping review synthesized evidence from 23 studies examining how tactile interaction translates physical phenomena—such as vibration, force, pressure, motion, and spatial structure—into perceivable sensory experiences that support conceptual engagement. Across the reviewed literature, tactile and haptic designs consistently function as sensory–representational learning systems through which learners explored scientific relationships through embodied interaction. The findings suggest that these approaches are most effective when integrated within coherent instructional environments that combine tactile interaction with conceptual explanation, teacher mediation, and complementary visual or signed resources. Although current evidence demonstrates stronger support for conceptual engagement than for validated skill transfer, the review highlights the importance of physics-informed design and pedagogical integration in advancing inclusive STEM learning environments. Future research should further examine how tactile learning environments support conceptual development, sustained participation, and transferable learning outcomes across diverse STEM learning contexts. Particular attention should be given to the rigorous evaluation of sensory–pedagogical design principles in inclusive educational settings.

## Data Availability

The original contributions presented in the study are included in the article/supplementary material, further inquiries can be directed to the corresponding author.
